# HISTOBREAST, a collection of brightfield microscopy images of Haematoxylin and Eosin stained breast tissue

**DOI:** 10.1038/s41597-020-0500-0

**Published:** 2020-06-05

**Authors:** Roxana M. Buga, Tiberiu Totu, Adrian Dumitru, Mariana Costache, Iustin Floroiu, Nataša Sladoje, Stefan G. Stanciu

**Affiliations:** 1 Center for Microscopy-Microanalysis and Information Processing, Politehnica University of Bucharest, Bucharest, Romania; 2 School of Life Sciences, Ecole Polytechnique Fédérale de Lausanne (EPFL), Lausanne, Switzerland; 3 Department of Pathology, Carol Davila University of Medicine and Pharmacy, Bucharest, Romania; 4 Department of Pathology, Emergency University Hospital, Bucharest, Romania; 5 Faculty of Medical Engineering, Politehnica University of Bucharest, Bucharest, Romania; 6 Centre for Image Analysis, Department of Information Technology, Uppsala University, Uppsala, Sweden; 7 Mathematical Institute of the Serbian Academy of Sciences and Arts, Belgrade, Serbia

**Keywords:** Image processing, Data acquisition, Microscopy, Breast cancer, Computational science

## Abstract

Modern histopathology workflows rely on the digitization of histology slides. The quality of the resulting digital representations, in the form of histology slide image mosaics, depends on various specific acquisition conditions and on the image processing steps that underlie the generation of the final mosaic, e.g. registration and blending of the contained image tiles. We introduce HISTOBREAST, an extensive collection of brightfield microscopy images that we collected in a principled manner under different acquisition conditions on Haematoxylin - Eosin (H&E) stained breast tissue. HISTOBREAST is comprised of neighbour image tiles and ensemble of mosaics composed from different combinations of the available image tiles, exhibiting progressively degraded quality levels. HISTOBREAST can be used to benchmark image processing and computer vision techniques with respect to their robustness to image modifications specific to brightfield microscopy of H&E stained tissues. Furthermore, HISTOBREAST can serve in the development of new image processing methods, with the purpose of ensuring robustness to typical image artefacts that raise interpretation problems for expert histopathologists and affect the results of computerized image analysis.

## Abstract (summary)

**Table:** 

Measurement(s)	H&E-stained fixed tissue slide specimen • breast carcinoma • histology images
Technology Type(s)	brightfield microscopy • histological assay
Factor Type(s)	Exposure • Gain • Gamma • magnification • image quality • image overlap • image mosaics
Sample Characteristic - Organism	Homo sapiens

Machine-accessible metadata file describing the reported data: 10.6084/m9.figshare.12240290

## Introduction

Over the past couple of decades medicine witnessed massive transformations and developments. In these efforts, the digitization of patient generated health-data has gained huge interest as it can enable sophisticated approaches for swift health state screening, or for automated analysis of complex multidimensional datasets for precise diagnostics^1–4^. Artificial Intelligence approaches, which have taken the field of medicine by storm in recent years, rely as well on digitized (big) medical data^5,6^. Furthermore, digital data can be relatively easily annotated^7^ and quickly retrieved from databases based on keyword queries, which can greatly facilitate its use for educational purposes^8–11^ or for correlative diagnostics assays^8,12–14^. The field of histopathology also benefits of these trends, making use of prominent advantages of the digital age, such as the portability of information and exponential growth of the computing power and its availability. An important step for exploiting these advantages has been made through the Whole Slide Imaging (WSI) approach, which can be used to scan an entire histology slide and convert it to a digital format. WSI facilitates medical data storing and manipulation^13,15^, together with cross-borders telemedicine^14,16,17^ and medical education^8,9,18^. Furthermore, WSI is useful to histopathologists to refine their decisions by enabling computer-aided diagnostic assays. These can automatically highlight diagnostics cues that can lead to higher diagnostic accuracy^19–21^, while also saving time.

Even though WSI is becoming widely spread, its use remains confined to countries with well-developed economies, due to the associated costs of the required hardware and software tools, and of data storage^15^. An alternative solution to WSI consists in the use of conventional light microscopes^22,23^ equipped with dedicated digital cameras (or even coupled to smartphone cameras^24,25^) in combination with algorithms for microscope image mosaicking (aka stitching), e.g.^26,27^. The mosaicking process consists in recording image tiles that overlap (by means of manual or programmable motorized stages) and then stitching these together to obtain the image mosaic of the entire histology slide (or of a part of it). Throughout the paper we will refer to such sets of image tiles that can be assembled to constitute a mosaic as Neighbour Image Tiles (NITs). The quality factor of the histology slide image mosaic (HSIM) depends on a series of underling image processing steps, such as registration and blending (known as *stitching*) of the contributing NITs^22,26^. In turn, the result of these operations depends on the properties of the NITs making up the HSIM, which depend on the acquisition conditions, such as contrast, brightness and others. Same as in image panoramas acquired for natural scenes^28^, building a HSIM (of the whole slide, or only of part of it) using NITs acquired under different acquisition conditions (e.g. illumination) can result in prominent image artefacts, known as image seams^29,30^. Such artefacts have a significant importance with respect to HSIM analysis, as they can cause improper or deficient interpretation of the final image by the human or the automated expert, with implications in the diagnostic accuracy. Given that image artefacts (or other quality related issues) can raise interpretation problems for expert histopathologists and affect the results of computerized image analysis, accurately assessing HSIM quality is very important.

Given the aforementioned context, there is a great need for datasets that can be used to evaluate, test, and benchmark the effects of various image processing operations required for HSIM assembly on the overall quality of the resulted images. At the same time, publicly available NITs and HSIMs collected with different quality attributes would also be of great benefit to the computer vision communities that develop image analysis algorithms for digital pathology. With such datasets, the developers can consider specific problems related to variations in HSIM quality, and hence evaluate and consolidate the robustness of their methods with respect to such issues.

To respond to these needs, we introduce here HISTOBREAST^31^, an extensive collection of brightfield microscopy (BM) images collected on a Haematoxylin - Eosin (H&E) stained breast tissue sample. HISTOBREAST is comprised of NITs collected at different acquisition settings and at two different magnifications, and also includes an ensemble of HSIMs composed from different combinations of the available NITs, exhibiting progressively degraded quality levels, according to a hierarchy that we previously introduced^32^. We envision that HISTOBREAST can be useful in the development and benchmarking of a variety of image processing and analysis algorithms/methods relevant for digital histopathology, such as image quality assessment, restoration, registration or mosaicking. In the following we present the structure of HISTOBREAST, the NITs acquisition and HSIM generation protocols, together with the interpretation of the imaged scenes, and discuss potential utility scenarios.

## Data Records

The HISTOBREAST^31^ collection is comprised of BM images acquired on a tissue fragment from a patient diagnosed with moderately differentiated (G2) invasive breast cancer of no special type (NST) with extensive foci of high grade ductal carcinoma *in situ* (DCIS)^33^. More specifically, HISTOBREAST consist of four Sets of NITs and two Sets of HSIMs, with each Set hosting multiple Subsets, which are further structured in Versions. The four NITs Sets are comprised of a total number of 1216 of image tiles, summing up ~35 GB. All image tiles have been recorded at a digital resolution of 3648 × 2736 pixels and are provided in Tagged Image File Format (TIFF). The two HSIMs Sets are comprised of a total number of 578 HSIMs, summing up 163 GB. All six HISTOBREAST Sets are accompanied by *ReadMe.txt* files describing their structure, which we also briefly detail next.

The four NITs Sets consist of NITs that were acquired under different exposure, gain and gamma settings, with 5x and 50x objectives, at two image tile overlap levels, ~15% and ~25%. The nomenclature of the HISTOBREAST folders hosting the four NITs Sets reflects these latter two conditions (magnification and overlap level):

- “5x_15per_overlap_tiles”
- “5x_25per_overlap_tiles”
- “50x_15per_overlap_tiles”
- “50x_25per_overlap_tiles”
  Each NITs Set contains three Subsets, named “Exposure”, “Gain”, “Gamma”. The nomenclature of the Subsets indicates thus the parameter modified during acquisition, as for each Subset a single parameter (exposure, gain or gamma) was modified, the other two being kept constant at a reference value. Furthermore, each Subset is structured in two Subset Versions, each one corresponding to an increase or a decrease of the modified parameter’s value, with respect to a reference value. The “Reference” Subset contains the NITs acquired under the reference setting (see ***Description of HISTOBREAST***).
  The nomenclature of the HISTOBREAST folders hosting the two HSIMs Sets is:
- “5x_25per_overlap_mosaics”
- “50x_25per_overlap_mosaics”

The two HSIMs Sets host HSIMs that were assembled from NITs acquired by individually varying the exposure, gain or gamma parameters. The varying range and values were specifically selected to allow achieving a known hierarchy among the generated HSIMs^32^. Same as for the NITs Sets, the HSIMs Sets are organized into three Subsets, “Exposure”, “Gain” and “Gamma”, according to the parameter modified within the stitched NITs. Each Subset is further structured in two Subset Versions according to an increase or decrease in the value of the parameter considered for generating the Subset (compared to the reference value). The filename syntax of a HSIM indicates via an integer value its quality level; a higher integer value is equivalent to lower HSIM quality and vice-versa. A fourth Subset coined “Reference” contains the HSIM assembled using the reference NITs (see ***Description of HISTOBREAST***). Along with the HSIMs we provide as registration ground truth the coordinates of the incorporated NITs.

In brief, in the HISTOBREAST^31^ collection root folders represent the Sets, level one folders represent Subsets, and level two folders represent Subset Versions.

## Description of HISTOBREAST

**Fig. 1:**
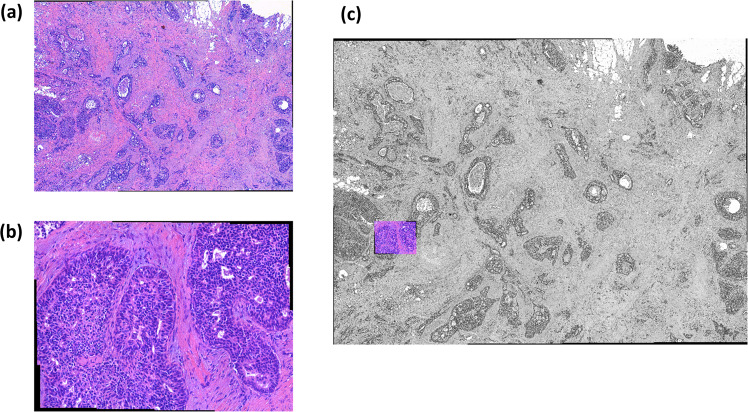
HSIMs of H&E stained breast tissue. The two HSIMs are assembled from image tiles collected with (**a**) a 5x magnification objective and (**b**) a 50x magnification obj. (**c**) Geometrical correspondence between the two HSIMs depicted in (**a**) and (**b**).

The HISTOBREAST collection is comprised of four NITs Sets, which allow the assembly of a vast number of HSIMs that exhibit various quality levels and aspects. The first two Sets consist of NITs collected with a 5x magnification objective under ~15%, and ~25% overlap. The two other NITs Sets are acquired with a 50x magnification objective, under the same levels of overlap of ~15% and ~25%. The 5x and 50x NITs collected at ~25% overlap can be stitched together to constitute HSIMs depicting the two scenes presented in Fig. 1. Noteworthy, the sample region corresponding to the 50x HSIM is included in the one corresponding to the 5x HSIM. The NITs collected with the two objectives under 15% overlap can be stitched together to constitute HSIMs covering sample areas larger than those represented in Fig. 1 (which include these). In the next part we present the protocols for assembling the NITs and HSIMs Sets and provide the histopathological interpretation of the imaged scenes.

### NITs Sets: acquisition protocol

**Fig. 2:**
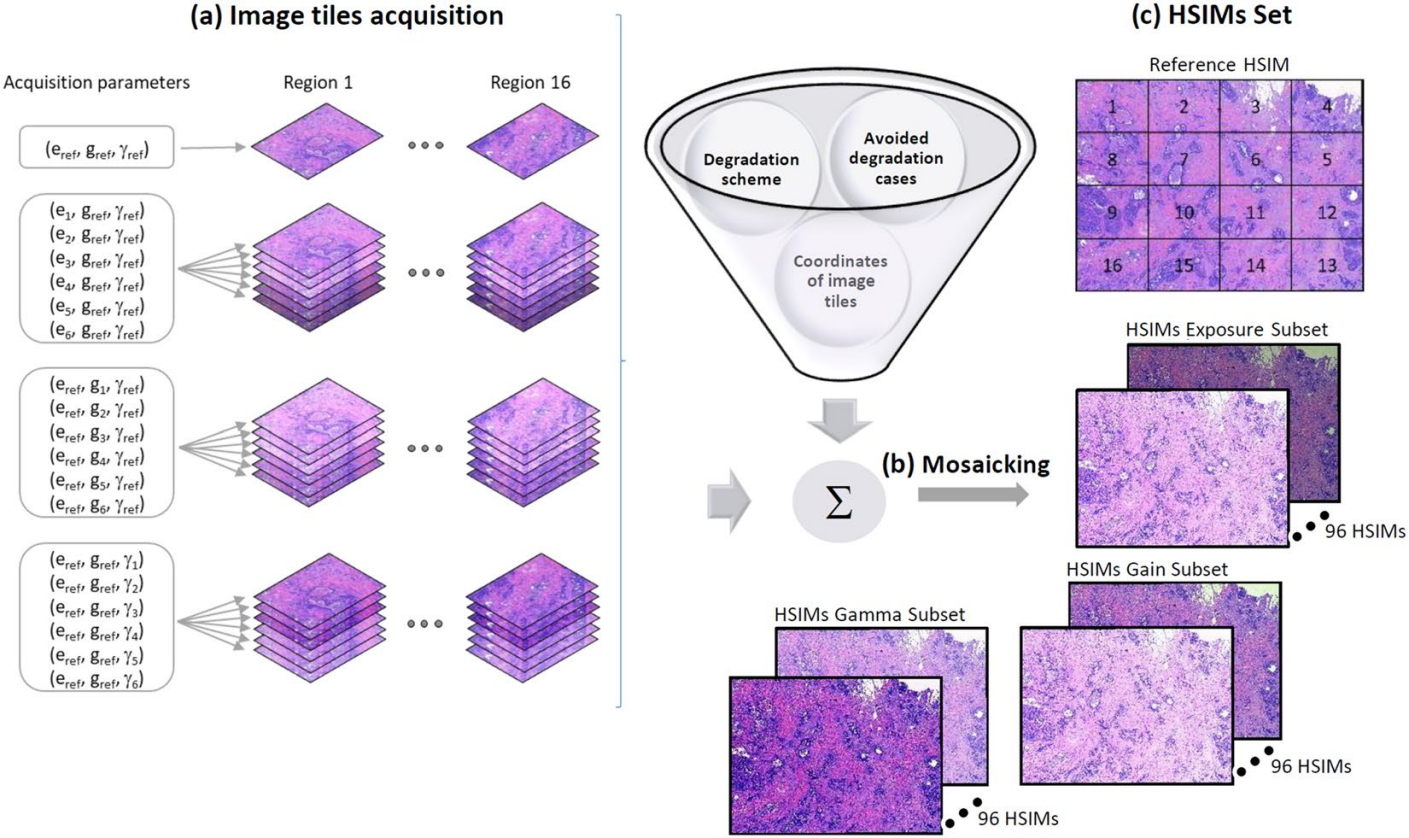
Acquisition protocol for generating the four NITs sets available in HISTOBREAST, together with an example of how these can be used to generate progressively degraded HSIMs. (**a**) Acquisition configuration for each of the 16 (overlapping) sample regions imaged to jointly constitute a geometrically homogenous HSIM (as depicted in Fig. 1a,b); 19 different acquisition settings were considered for each region (resulting thus in 19 versions of the same image tile) (**b**) Example scheme for generating HSIMs with various quality levels and aspects^32^. (**c**) The four NITs Sets are accompanied in HISTOBREAST by several collections of progressively degraded HSIMs generated using the available image tiles and the previously proposed controlled degradation scheme^32^.

The NITs Sets were assembled by imaging 16 neighbour sample regions, for each of these regions 19 images being collected under different acquisition conditions, Fig. 2a. The parameters that have been modified to generate these 19 image tile variants are: exposure time (e), gain (g) and gamma (γ). These three parameters have the following interpretations:

- Exposure time represents the length of time when the s-CMOS sensor is exposed to light
- Gain represents an image acquisition setting by which the amplification of the whole signal (including associated background noise) from the camera sensor is controlled
- Gamma represents a digital camera setting that controls the grayscale reproduced in the image; a gamma value equal to unity indicates that the camera sensor exhibits a linear response and thus precisely reproduces the object’s grayscale.

We considered these three parameters as they are the main to require a recalibration before acquiring each of the required NITs in a typical HSIM assembly protocol. This recalibration procedure is needed to achieve a homogeneous HSIM with respect to features such as contrast, brightness, sharpness, etc. Different calibration methods, and the use of different mosaicking algorithms, can result in HSIMs with distinct quality levels.

Each NITs Set is structured in four Subsets, reflecting the acquisition parameters. These Subsets are discussed next:

#### The “Reference” Subset

In the adopted approach for NITs acquisition and HSIM assembly, for each considered sample region an image tile was recorded under an acquisition configuration identified by two expert pathologists involved in this study as being optimal with respect to the visibility of diagnostic features. Under this configuration, specific values of the exposure time (e), gain (g) and gamma (γ) settings were selected so that the recorded images exhibit a good contrast between the two markers used for staining, Haematoxylin and Eosin. We further refer to these optimal acquisition parameters as e_ref_, g_ref_, γ_ref_, and to the corresponding images as *reference parameters* and *reference image tiles*, respectively. Within each of the four NITs Sets the *reference image tiles* constitute the “Reference” Subset. To support our claim that the *reference parameters* were selected such that the imaged scenes (interpreted later) are optimally visualized, we find important to mention that: (i) a strong contrast between Haematoxylin and Eosin is very useful for the study of desmoplastic stroma or collagen-rich areas (including some vessels hyalinization), (ii) a strong contrast between Haematoxylin and Eosin highlights the clear optical spaces of the adipose tissue and the luminal component of the ductal carcinoma *in situ*, especially of low-grade appearance with a cribriform pattern, (iii) optimal brightness favours the success of detecting cellular and nuclear details that are relevant for establishing a correct Elston-Ellis grading score.

#### The “Exposure”, “Gain” and “Gamma” Subsets

The NITs in each of these Subsets were acquired by varying the acquisition parameter which gives the name to the respective Subset (e.g. the exposure parameter was varied for generating the “Exposure” Subset), while keeping the other two imaging parameters fixed, at their reference value. The acquisition parameter that was varied to generate a NITs Subset takes six values, in addition to the reference value (e.g. e_ref_ e_1_, e_2_, e_3_, e_4_, e_5_, e_6_ for the “Exposure” Subsets), as presented in Table 1. The step to vary each parameter was chosen so that the differences between image tiles are consistent, while ensuring that the extreme values of the parameters’ range result in image tiles whose quality level is not fully compromised, and still meaningful for diagnosis. We refer to the image tiles acquired under non-reference parameters as *degraded tiles*, as they exhibit a sub-optimal aspect. The numerical values corresponding to each parameter used in the acquisition process are provided in Table 1.

**Table 1:** The acquisition parameters used for generating the NITs Sets.

Exposure parameter	e_6_	e_5_	e_4_	e_ref_	e_1_	e_2_	e_3_
Value [ms]	2.60	3.60	4.70	5.70	6.60	7.50	8.60
**Gain parameter**	**g**_**6**_	**g**_**5**_	**g**_**4**_	**g**_**ref**_	**g**_**1**_	**g**_**2**_	**g**_**3**_
Value	1.2	1.4	1.6	1.8	2	2.2	2.5
**Gamma parameter**	**γ**_**6**_	**γ**_**5**_	**γ**_**4**_	**γ**_**ref**_	**γ**_**1**_	**γ**_**2**_	**γ**_**3**_
Value	0.45	0.50	0.55	0.60	0.70	0.80	1

The NITs Subsets were further organized in six Subset Versions (two per each Subset), reflecting unidirectional variations of the considered parameters, as follows:

- Exposure increase: (e_1_, e_2_, e_3_) • Exposure decrease: (e_4_, e_5_, e_6_)

- Gain increase: (g_1_, g_2_, g_3_) • Gain decrease: (g_4_, g_5_, g_6_)
- Gamma increase: (γ_1_, γ_2_, γ_3_) • Gamma decrease: (γ_4_, γ_5_, γ_6_)

To conclude on this part, to generate a NITs Set 16 sample regions were imaged, each under 19 distinct acquisition settings (Table 1), resulting in 304 image tiles per NITs Set. The four NITs are thus comprised of a total of 1216 image tiles. These can be used independently, or as assembled HSIMs, to benchmark image processing and analysis methods relevant for digital pathology (see ***Utility***).

### HSIMs Sets: generation protocol

Using various combinations of image tiles collected at different acquisition settings one can generate HSIMs with a priori known quality levels or of random quality. These can be used in the development and benchmarking of image processing methods relevant for histopathology, such as image quality assessment, flat field correction, etc. (see ***Utility***)*.* To exemplify HISTOBREAST’s usefulness in this regard, we provide two Sets of HSIMs with progressively degraded quality. Each HSIMs Set corresponds to one of the two considered magnifications (5x and 50x), and both rely only on the NITs that we collected with 25% overlap. Obviously, similar HSIMs Sets can be generated by using the two other NITs Sets available in HISTOBREAST, collected at 15% overlap.

**Fig. 3:**
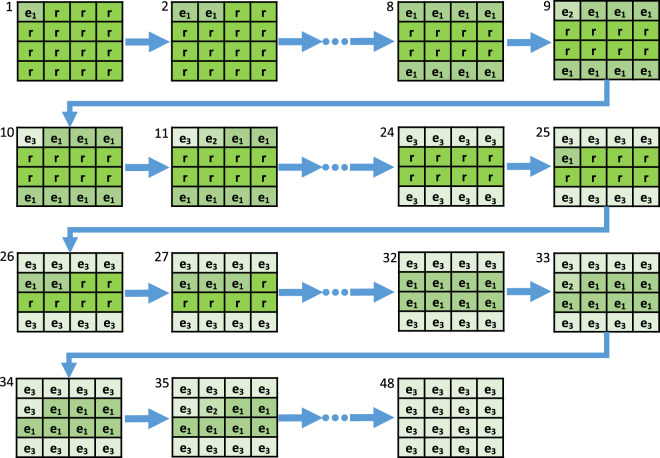
Representation of the HSIM degradation flow with the associated HSIM index and acquisition parameters for each image tile, in the case of “Exposure increase” Subset Version. Similar strategies were adopted for the other Subset Versions. HSIMs’ indices indicate their quality, according to the methodology presented in^32^ (higher index value indicates lower HSIM quality).

**Fig. 4:**
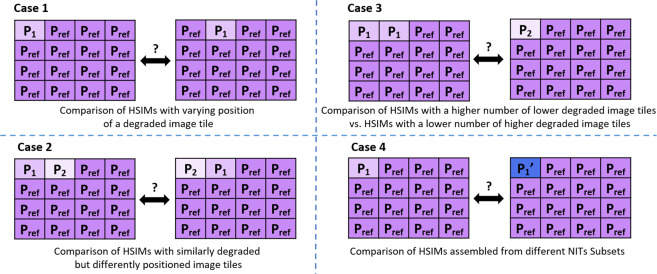
Examples of excluded HSIM generation scenarios, due to incompatibility with objective quality assessment.

The two HSIMs Sets are organized into three Subsets (“Exposure”, “Gain” and “Gamma”). The three Subsets contain instances of progressively degraded quality of the same HSIM, according to the acquisition parameters giving the name to the respective Subset. Furthermore, the three Subsets are organized in six Subset Versions (two per Subset), whose structure is the same with that of the NITs Subset Versions (Exposure increase/decrease, Gain increase/decrease, Gamma increase/decrease). The progressively degraded HSIMs available in these Subset Versions were generated using the strategy described in our previous work^32^ and depicted in Fig. 2. The principle of this progressive degradation can be overviewed in Fig. 3, where we present a sequence of 48 progressively degraded HSIMs generated to constitute the “Exposure increase” Subset Version in a HSIMs Set. A similar strategy is adopted to assemble all Subset Versions in the HSIMs Subsets. As can be observed in Fig. 3, each Subset Version satisfies a unidirectional variation of the modified parameter. This approach was adopted to simplify the comparison of HSIMs in terms of their quality, and hence ensure their correct ranking. Representative images belonging to some of these Subset Versions are presented in our previous work^32^, where several cases which could lead to a questionable objectivity with respect to HSIM quality assessment were also identified (examples are shown in Fig. 4). The HSIMs corresponding to these ambiguous cases have been left out of HISTOBREAST, but, if needed, the user can easily generate them. In each HSIMs Set, together with the “Exposure”, “Gain” and “Gamma” Subsets we provide a reference HSIM constituted of NITs collected at the reference settings, which can be regarded as ground-truth in terms of HSIMs quality.

### Interpretation of the imaged scenes

In the context of an invasive breast cancer of no special type, the HSIM assembled from the reference NITs collected at 5x magnification (Fig. 1a) shows an invasive component exhibiting sheets, nests, cords or individual malignant cells with prominent ductal-tubular differentiation. The overall Elston-Ellis score is 6: glandular acinar/tubular differentiation in about 50% of tumoral areas – score 2; the score for nuclear pleomorphism is also 2 as the nuclei are larger than normal with visible nucleoli, and moderate variability in both size and shape and finally the score for mitotic figures is 2 as the mitotic count revealed approx. 12 mitotic figures, 10 high power fields (HPF), field diameter 0.65 mm. As such, the invasive tumoral proliferation can be regarded as moderately differentiated (G2). The tumour exhibits an extensive *in situ* component, mainly high grade, but also some foci of low-grade DCIS with predominantly a cribriform pattern. The *in situ* component also shows comedo-type necrosis and microcalcification, however the diagnosis of high grade DCIS is based on the nuclear features of the lesions, and not by the presence of the comedo-type necrosis; even if this latter feature is commonly seen with high-grade DCIS, its presence is not obligatory^33,34^. In addition, a desmoplastic stromal reaction can also be observed in Fig. 1a, which obscures the tumor cells in some areas. The dense collagenous stroma had apparently few stromal cells, but focally some inflammatory cells (tumor infiltrating lymphocytes - TILs) can be observed, especially at the periphery of the infiltrative component of the lesion.

The HSIM assembled from the reference NITs collected at 50x magnification (Fig. 1b) shows typical features of florid ductal hyperplasia: the epithelial cells almost completely fill the ducts but with fenestrations and slits spaces (irregular pseudolumina at periphery of the lesion). Some important features to highlight are: overlapping neoplastic cells with indistinct borders, acidophilic and granular cytoplasm, and oval normochromatic nuclei with small or indistinct nucleoli. There are no mitotic figures or necrotic areas. At the periphery of the lesion elongated myoepithelial cells and few macrophages are present. The adjacent stroma has a few chronic inflammatory cells (lymphocytes). This relatively common breast lesion has a 1.5 to 2 time increased risk for invasive carcinoma. We find noteworthy to highlight in the context of the scene imaged in Fig. 1b, that florid ductal hyperplasia may evolve into a true precursor lesion: *in situ* ductal carcinoma. In fact, low-grade ductal carcinoma *in situ* is the main differential diagnosis to be considered. When the lesion develops into a ductal carcinoma *in situ* the pseudolumina become more rounded with a rigid appearance (Roman bridges) and the cell population is more monotonous, polarized toward luminal space, the ratio between the nucleus and the cytoplasm is slightly increased and mitotic figures are more common.

We find relevant to mention that while high-magnification (Fig. 1b) is important for assessing the morphology of cellular components relevant in the diagnostics of breast cancers (e.g. nuclei), low magnification (Fig. 1a) is also very important for the histopathological characterization of breast cancers, representing the most convenient method for documenting and assessing the histomorphological prognostic parameters (i.e., the distribution of the tumour’s invasiveness and *in situ* components, tumor extension -including tumor size, pattern of invasion, presence of calcification, desmoplasia and necrosis and even subgross evaluation of the therapeutic effect post neoadjuvant therapy). Typically, low magnification (5x or 10x magnification objectives) is used in conjunction with systematic radiological-pathological and clinical data.

## Utility

As described in the previous section, the HISTOBREAST collection is comprised of four NITs Sets, and two HSIMs Sets. All the provided NITs and HSIMs, as well as the vast number of additional HSIMs that can eventually be generated based on the available data, are in our opinion useful in the development and benchmarking of various image processing and analysis methods relevant for digital histopathology. With respect to the additional HSIMs that can be generated based on the available NITs, we refer here to the “Exposure” NITs Subset which contains 7 image tile versions for each of the 16 regions. Thus, 7^16^ (3.3232931e + 13) different HSIMs can be assembled, exhibiting various quality levels (either progressively increasing/decreasing or random) and appearances. On the other hand, if we would consider all the image tiles available in a NITs Set (for each tile, 3 parameters are modified with 6 different degradation levels each, plus the reference value), then a total number of 19^16^ (2.8844141e + 20) different HSIMs can be assembled. Furthermore, if we consider all image tiles available within the four NITs Sets, this number would be 4 times larger (1.1537656e + 21). The potential usefulness of this vast number of HSIMs that can be generated is augmented by the fact that HISTOBREAST NITs and HSIMs are accompanied by two types of annotation/ground truth: (1) true positions of the NITs assembled in a HSIM, and (2) knowledge of optimal image quality (for both NITs and HSIMs). All these enable the utilization of HISTOBREAST in the development and benchmarking of methods and tools for a series of applications that we discuss next.

### Image registration and stitching

*Image registration* is the process of bringing in the same system of coordinates, and geometrically aligning, two or more images of the same scene, which may be taken at different times, under different acquisition conditions, or by different sensors^35^. In microscopy imaging, multimodal and/or monomodal registration are typically required to perform side-by-side image comparison, which is often needed to monitor morphological changes and motion, or to perform template-, or atlas-based segmentation, classification, detection, etc^36–41^. Furthermore, monomodal registration, e.g.^22,25,42^, is required for obtaining images that depict large sample areas, that cannot be imaged at the desired resolution with the imaging system’s maximum field-of-view (e.g. a FOV of 1 cm^2^ cannot be imaged in a single shot with a 100x objective). The HISTOBREAST collection contains four Sets, each comprised of 19 image tile variants (collected under different acquisition conditions) collected for 16 samples regions. Given the available ground-truth in terms of image tile coordinates, pairwise or group registration algorithms can be evaluated on any combination of the 19 corresponding image tiles (where overlap is available), for each of the 16 regions. In particular, the robustness of such registration algorithms to automatically calculate homographies between image tiles collected under varying conditions can be observed and evaluated based on the available NITs Sets. By including NITs of different levels of overlap, HISTOBREAST supports the evaluation of image registration algorithms with respect to robustness to overlap variation. In the case of HSIMs assembled based on manually collected neighbouring tiles, such variations are common. By including NITs collected on a particular sample region at two different magnifications (5x and 50x), HISTOBREAST supports the evaluation and development of multi-scale registration or patch/image matching and retrieval approaches.

Besides registration, another important operation required to perform image stitching in the purpose of assembling HSIMs from NITs consists in the blending of their overlap regions. This step is required to remove brightness differences at the NITs borders and ensure seamless integration. HISTOBREAST is thus also useful to evaluate various blending algorithms with respect to their capacity to provide seamless mosaics in the case of H&E images.

As an example on the dimension of our data set with respect to potential use in the development/benchmarking of registration/stitching methods, we can observe that for a pair of two overlapping tiles, and 19 versions of each of the two tiles, one can generate a total number of 19^2^ = 361 image pairs. For a 16 × 16 mosaic, we have 3 pairs of overlapping image tiles per each row, and 3 per each column, hence a total of 18 pairs. Therefore, the number of image pairs that can be registered/stitched available in HISTOBREAST is 18 × 361 = 6498 (without taking into account image pairs that overlap in diagonal direction, which are also available).

### Image quality assessment

Current Image Quality Assessment (IQA) methodologies are split in two main categories: subjective and objective approaches. While the former are based on the quality scores provided by human experts, the latter rely on mathematical models that can automatically provide an estimate over the perceived image quality (which aims to be consistent with that of a human observer). These objective methods are also divided into three main classes according to the availability of a distortion-free reference image: (i) No-Reference IQA (NR-IQA), a.k.a. “blind”, (ii) Reduced-Reference IQA and (iii) Full-Reference IQA (FR-IQA). An example on the utility of the HISTOBREAST image datasets consists in our previous work^32^, where we used part of HISTOBREAST’s progressively degraded HSIMs (those collected under 5x magnification) to benchmark a set of Full Reference Image Quality Assessment Algorithms. The evaluation of “blind” image quality assessment algorithms^43^ is also possible, considering that the quality level of each progressively degraded HSIM in HISTOBREAST is a priori known.

Furthermore, as discussed in the beginning of this section, exploiting all possible combinations of the image tiles available in HISTOBREAST, one can potentially generate a total number of 1.1537656e + 21 distinct HSIMs. These can be useful in the development/benchmarking of image quality assessment methods (reference based and blind).

### Image restoration and enhancement

Image restoration is the process of recovering an original image from its available degraded version. Availability of minimally degraded images (acquired by using the *reference parameters*), as well as availability of ordered sequences of degraded images, is essential for systematic evaluation of image restoration methods. The NITs and HSIMs contained in HISTOBREAST can be utilized to evaluate the performance of various image restoration methods, in particular of those focused on addressing exposure, gain, and gamma aspects. These parameters affect the brightness, the level of noise, and the amount of contrast present in the images.

Image enhancement techniques aim at emphasizing particular features of interest or at making images more visually appealing and, more importantly, easier to interpret, without utilizing any image formation model. They typically include image denoising^44^, contrast enhancement^45,46^ and flat-field correction methods^47,48^. The HSIMs available in HISTOBREAST, assembled from NITs systematically acquired under various acquisition settings, can be used for the development and benchmarking of image enhancement algorithms suitable for brightfield microscopy and histopathology.

### Evaluation of image analysis tools w.r.t. robustness under image degradation

A large number of image analysis methods frequently used in microscopy such as, image segmentation, feature extraction, feature description, object detection, and image classification are highly sensitive to image quality^49,50^. This is because the building pieces of such methods, e.g. salient points^51^, keypoint descriptors^52–54^, or texture features^55,56^, aim at capturing subtle intensity variations, hence, aspects such as artefacts, noise or contrast deficiencies^57^ usually interfere with their successful use. Furthermore, the performance of some of these methods holds deep implications for further analysis steps. Let us take, for example, image segmentation methods that aim to extract specific objects of interest from a certain scene/image for further analysis. These are typically based on intensity homogeneity (detecting homogeneous regions, or borders between them), thus are often highly sensitive to noise or contrast. Their success clearly affects all the subsequent results, e.g. the classification of the detected objects. It is therefore of highest importance to develop algorithms robust to variations in image quality and to evaluate their performance on images sets relevant for such tasks. HISTOBREAST can offer significant support in the development/benchmarking of image analysis tools, such as those above discussed. The availability of distinct variants of the same scene/sample region offers the possibility to experiment with the most frequently appearing types of image quality inconsistencies/problems in BM and evaluate the robustness of various algorithms w.r.t. them. HISTOBREAST not only offers a large number of NITs and HSIMs that are collected/assembled in a principled manner, but also enables the user to generate a vast number of additional HSIMs that can support other specific development/benchmarking purposes.

## Methods

### Instrumentation

The NITs provided in HISTOBREAST were collected with a Leica DM 3000 LED brightfield microscope, equipped with an MC 190 HD camera hosting an s-CMOS sensor with reduced noise factor^58^. For image acquisition we used two different magnifications 5x and 50x, available with HC PL Fluotar 5x/0.15 and N Plan L 50x/0.5 objectives. During image acquisition the field diaphragm was tuned to avoid vignetting effects. The digital resolution of the images collected in this work (with both objectives) is 3648 × 2736 pixels. For the 5x magnification obj. the pixel size and field of view are 0,60727271 μm and 2215.55 × 1661.50 μm, respectively, while for the 50x magnification obj. the pixel size and field of view are 0.060727271 μm and 221.53 × 166.15 μm, respectively.

### Assembly of HSIMs from NITs

All HSIMs provided in HISTOBREAST were generated using the Grid/Collection stitching plug-in^59^ from Fiji^60^, under the default settings. To ensure consistent alignment of the NITs in the assembled HSIMs we performed two steps. The first was to assemble the reference HSIM (from NITs collected at the reference parameters) using the sub-pixel accuracy option. The position of each image tile in the assembled reference HSIM was obtained by applying the stitching algorithm to the reference image tiles, and by utilizing a priori knowledge of the acquisition geometry (snake-by-rows with Right & Down orientation). The recovered coordinates of the reference tiles (provided in HISTOBREAST to serve as NITs registration ground-truth) were then further used in the second step, to assemble again the reference and all the degraded HSIMs (available in the “Exposure”, “Gamma” and “Gain” Subsets), without the usage of sub-pixel accuracy option. To alleviate image seams in the assembled HSIMs, the stitched image tiles were fused by the linear blending algorithm available in the above mentioned Fiji plugin.

## Technical Validation

All 19 variants available for each considered image tile/sample region were acquired in the same ambient light conditions. Furthermore, to validate that no sample drift had occurred while recording the required image tile variants of a particular sample region, a registration algorithm^61^ based on the cross-correlation function, implemented in the frequency domain by the use of Discrete Fourier Transform (DFT), was applied on each degraded tile. This algorithm was developed for recovering 2D translations and allows sub-pixel precision in the alignment of registered images. Usually, the DFT introduces typical errors (such as picket-fence), the translation invariant normalized RMS error having a non-zero value, this being reflected in some modified pixels’ values. In this case the pixels’ modification does not exceed unity, resulting in no changes within the registered tiles when compared to the degraded ones. After assessing the output error of the algorithm obtained between the reference tile and the registered one, no row or column shift was reported.

### Sample preparation and interpretation

Specimen samples were fixed with 10% buffered formalin for 24 hours and were processed by conventional histopathological methods using paraffin embedding, 3 µm thick sectioning followed by H&E staining. The grade of the tumor was established using the Elston-Ellis grading system (Nottingham modification of Bloom-Richardson system).

### Ethics statement

The human tissue samples imaged in this experiment to generate the introduced dataset were available to the authors in the frame of the PN-III-P2-2.1-PED-2016-1252 MICAND grant funded by the Romanian Executive Agency for Higher Education, Research, Development and Innovation Funding (UEFISCDI). In this research grant, run in collaboration by Politehnica University of Bucharest and the Carol Davila University of Medicine and Pharmacy, the rules in effect for obtaining patient informed consent, established by the Ethics Committees of the two institutions, were fully respected by the researchers involved in this study.

## Data Availability

The Grid/Collection stitching plug-in^59^ of Fiji used for generating the HSIMs collections is publicly available as a Java Script.
